# Who consumes curative care expenditure of medical institutions in Beijing: a case study based on System of Health Accounts 2011

**DOI:** 10.1186/s12913-023-09564-8

**Published:** 2023-05-25

**Authors:** Yan Jiang, Xiaowei Man, Xuefeng Shi, Liying Zhao, Wanjin Yang, Wei Cheng

**Affiliations:** 1 Beijing University of Chinese Medicine, No.11 North 3Rd Ring Road East, Chaoyang District, Beijing, China; 2 National Institute of Chinese Medicine Development and Strategy, Beijing, China

**Keywords:** Curative care expenditure, Medical institutions, System of Health Accounts 2011, Disease system, Distribution

## Abstract

**Background:**

China’s health system is challenged by complex health problems experienced by different population groups and caused by multiple diseases. This study examined the distribution of curative care expenditure (CCE) of medical institutions in Beijing using beneficiary characteristics such as residency, gender, age, and disease. Suggestions are presented for the development of health policies.

**Methods:**

A total of 81 medical institutions with approximately 80 million patients in Beijing, China, were selected via a multistage stratified cluster random sampling approach. Based on this sample, the System of Health Accounts 2011 was used to estimate the CCE of medical institutions.

**Results:**

The CCE of medical institutions in Beijing was ¥246.93 billion in 2019. The consumption of patients from other provinces was ¥60.04 billion, accounting for 24.13% of the total CCE. The CCE of female consumption (52.01%/¥128.42 billion) exceeded that of male consumption (47.99%/¥118.51 billion). Almost half of the CCE (45.62%/¥112.64 billion) was consumed by patients aged 60 or above. Adolescent patients up to an age of 14 (including those aged 14) mainly chose secondary or tertiary hospitals for treatment. Chronic non-communicable diseases accounted for the largest share of CCE consumption, with circulatory diseases accounting for the highest proportion.

**Conclusions:**

This study identified significant differences in CCE consumption in Beijing according to region, gender, age, and disease. Currently, the utilization of resources in medical institutions is not reasonable, and the hierarchical medical system is not sufficiently effective. Therefore, the government needs to optimize the allocation of resources according to the needs of different groups and rationalize the institutional process and functions.

## Background

As in other developing countries, the increasing number of chronic non-communicable diseases in China is a major health problem [1, 2]. Moreover, certain existing or emerging infectious diseases, such as COVID-19, are also posing serious threats to China's health system. Therefore, increasingly limited medical resources must suffice to cope with pressures caused by chronic non-communicable diseases and infectious diseases in China [3–6].

China is also an aging society, where the proportion of the elderly (aged 65 years of above) reached 11.79% in 2020. While the elderly have become the main consumers of medical resources, the health problems of adolescents also deserve more attention. Diseases including myopia, obesity, and psychological illness have become major threats to adolescents [5, 7–9].

China’s health system is currently facing various problems caused by different groups and multiple diseases and has to deal with health problems increasing in complexity and covering all ages [10]. The first national medium- and long-term strategic plan for population health (i.e., Healthy China 2030 Plan) highlights the need to adjust and optimize the health service system. This plan focuses on solving the health problems of key population groups such as women, children, the elderly, and the floating population, with the goal to better meet the medical needs of different patients [11]. The hierarchical medical system (HMS) refers to the grading of diseases according to their priority and ease of treatment, with medical institutions at different levels undertaking the treatment of different diseases. HMS has been established gradually with the purpose to improve the efficiency of medical resources and maximize the satisfaction of multiple medical needs of various Chinese groups. On the one hand, HMS can reduce the curative care expenditure (CCE) of patients with common and chronic diseases by guiding the flow of this group of patients to primary medical institutions [12, 13]. On the other hand, HMS can also prevent the occurrence and development of diseases through the prevention and management of chronic diseases at the level of primary medical institutions. This can be achieved by strengthening the management of chronic diseases, which can prevent excessive growth in CCE and improve the efficiency of the utilization of health resources in the long run [14]. Previous studies on HMS mainly focused on its advantages, implementation, and results. Li (2019) presented a framework to solve the problem of "downward-referral" through classifying hospitals at different levels by professional doctors [15]. Zhang (2018) proposed a framework to classify the different degrees of diseases according to the diagnosis by a doctor, which was a key step in promoting the HMS [16]. Zhou (2021) evaluated the effect of HMS on the health-seeking behavior of Chinese patients using panel data [17].

However, little research has focused on the population differences between patients treated by medical institutions at different levels. It is of great importance to examine the conditions of patients of all ages and various diseases served by medical institutions at all levels. Such an examination can provide sufficient data support for policy makers to formulate scientific policies and arrive at a more reasonable allocation of medical resources.

As relevant evidence has not been reported to date, this study attempts to fill the knowledge gap by analyzing the results of the System of Health Accounts 2011 (SHA 2011). The distribution of CCE is accessed using primary data collected from health institutions and secondary data obtained from National Health Accounts or other sources. As the capital of China, issues with the health system (such as an aging population and high prevalence of chronic diseases) are more pronounced in Beijing. Chronic diseases are among the top 10 reasons why Beijing residents die, and the mortality rate of chronic diseases reached 645.7 per 100,000 in 2020 [18]. Survey data have shown that the prevalence of chronic diseases among Chinese people over 65 years old was 623.3‰ in 2018, which is much higher than the population average of 342.9‰ [19]. While the proportion of the resident population over 65 years old in Beijing reached 13.40% in 2020, which was higher than the national rate of 11.79%. Beijing also has the highest health expenditure per capita in China [20]. To alleviate the contradiction between available health resources and health expenditure, Beijing has carried out two important rounds of medical price reforms over the past 5 years. These are named the Comprehensive Reform of the Separation of Medicines and the Comprehensive Reform of the Medical Consumption Linkage [21, 22]. As the reform continues to deepen, the difficult step is to carry out accurate medical resource allocation matching the characteristics of medical resource consumption of different populations. Accurately allocating these resources can improve the accuracy of reform decisions. To provide the basis for the formulation of and decision-making on the strategies of Beijing’s future medical reforms, it is necessary to identify the consumers of CCE in different medical institutions. This research also presents a useful exploration for the precise allocation of medical and health resources in developing countries.

## Methods

### Definition

In this study, the CCE of medical institutions refers to the expenditure of therapeutic supplies and services consumed to restore, maintain, and improve the health condition of patients in Beijing's medical institutions over one calendar year. Unlike the conventional caliber of SHA 2011, in this study, the CCE of medical institutions includes both the CCE of the residential population in Beijing and the CCE generated by external patients at medical institutions located in Beijing. In Beijing, local residents are permanent residents. The CCE includes expenditure on outpatient care and inpatient care [23]. In this study, medical institutions include all hospitals and community hospitals.

### Data source

In this study, information was drawn from both primary and secondary data. First, officially published data on revenues and various subsidies as well as service volumes for all healthcare institutions in Beijing in the year 2019 were collected. The sources were the Beijing Health Financial Statistical Yearbook 2020, the Beijing Health Statistical Yearbook 2020, and the Beijing Health Accounts Report 2020. These data were used to estimate the total CCE. The data related to the revenue and service volume of governmentally run public hospitals were obtained from the Beijing Health Financial Statistical Yearbook 2020. The data related to non-governmentally run hospitals were obtained from the Beijing Health Statistical Yearbook 2020. Data on financial subsidies and health insurance reimbursements were obtained from the Beijing Health Accounts Report 2020. Second, the survey instruments were designed to collect primary data from various medical institutions in Beijing to obtain allocation parameters for the analysis of CCE based on the characteristics of beneficiaries.

### Sample institutions

In China, medical institutions can be classified into central-, provincial/municipal-, county/district-, and community-level hospitals according to the level of government they are affiliated with. Alternatively, they can be classified according to the type of ownership into public or socially run medical institutions. Central hospitals are uniformly managed by the National Health Commission. Municipal hospitals are managed by the Beijing Hospital Administration. District- and community-level hospitals, as well as social-run hospitals are administered by the health commissions of districts in Beijing.

Given the great differences between municipal hospitals in Beijing, a total of 22 institutions were sampled. For district-level and community-level institutions, a multi-stage sampling method was adopted. In the first stage, four of the 16 districts in Beijing (i.e., Dongcheng, Fengtai, Changping, and Pinggu) were selected based on principal component analysis, and five indicators were included (i.e., financial subsidy income, number of health technicians, per capita GDP, per capita government health expenditure, and permanent resident population density). In the second stage, 20% of streets in each district were randomly selected. In the third stage, one hospital was randomly selected in each district and one community hospital was randomly selected on each street. Moreover, to reflect the spending of private hospitals, 13 socially run hospitals were also selected based on their service characteristics, representativeness, and information quality. Considering the representativeness of the sample and the availability of data, central hospitals were not included in the sample.

A total of 81 institutions were examined, including 39 public hospitals, 13 socially run hospitals, and 29 community hospitals. From the surveyed institutions, the records of about 80 million patients were obtained, including data from January 1, 2019, to December 31, 2019. These data include information on age, gender, primary diagnosis, residency (Beijing resident or not), and cost after desensitization of personal information.

According to the grading standard, institutions are divided into community hospitals (first level), secondary hospitals (second level), and tertiary hospitals (third level). Community hospitals provide health care services for the residents of specific communities; secondary hospitals provide comprehensive medical and health services to multiple communities; tertiary hospitals provide high-level, specialized medical and health services nationwide to treat intractable diseases [24, 25]. In addition, hospitals can also be divided into various types of health institutions, such as general hospitals, hospitals practicing traditional Chinese medicine (TCM), and specialty hospitals.

## Methods

SHA 2011 is universally acknowledged as a system of total health expenditure accounting, which is suitable for cross analyzing regional health expenditure, including source, institution, and service utilization [23, 26]. It is mainly used to study national or regional healthcare financing schemes and healthcare functions [27, 28]. In recent years, researchers have also begun to examine the cost of different age groups or disease groups in different countries or regions [29, 30].

To obtain the total CCE and its disaggregation by beneficiary characteristics, a top-down approach was used. In the first step, the total CCE of all medical institutions in Beijing was measured by different levels and types through secondary data obtained from official sources. In the second step, the composition of CCE by gender, age, and disease was estimated for different levels and types of medical institutions in the same year from the collected primary data. In the third step, the proportional estimates obtained in the second step were applied to the total CCE for the different levels and types of healthcare facilities accounted for in the first step. Finally, the composition of CCE by gender, age, and disease in different levels and types of medical institutions was obtained. The underlying assumption of this strategy was that the estimates derived from the four selected districts are representative at the municipal level.

Taking the hospital as an example, how CCE was derived from beneficiary characteristics can be illustrated. Firstly, the CCE of hospitals at the level *a* type *b* in 2019 was accounted for, which was named CCE_*ab*_. Secondly, using data retrieved from the Hospital Information System of sampled hospitals at the level *a* type *b* in the same year (2019) enabled to generate percentages of outpatient or inpatient spending of the total CCE based on gender, age, residency, and disease using primary diagnostic information. For example, the percentage of the cost for local (*l*) outpatients (*o*) with respiratory system diseases (*h*) in the male (*m*) age group under 5 years old (*k*) in level *a* type *b* sampled hospitals was named P_*abolkmh*_. The accounting of the CCE for respiratory diseases in males under 5 years old in local outpatients of level *a* type *b* hospitals in Beijing (which was named CCE _*abolkmh*_.) was multiplied by the CCE_*ab*_ of P_*abolkmh*_ [27, 28, 31].

$${\mathrm{CCE}}_{abolkmh.} = {\mathrm{CCE}}_{ab }\times {\mathrm{P}}_{abolkmh}$$

## Results

### Distribution of CCE according to region, gender, age, and disease system

#### Composition of CCE in different regions

**Fig. 1:**
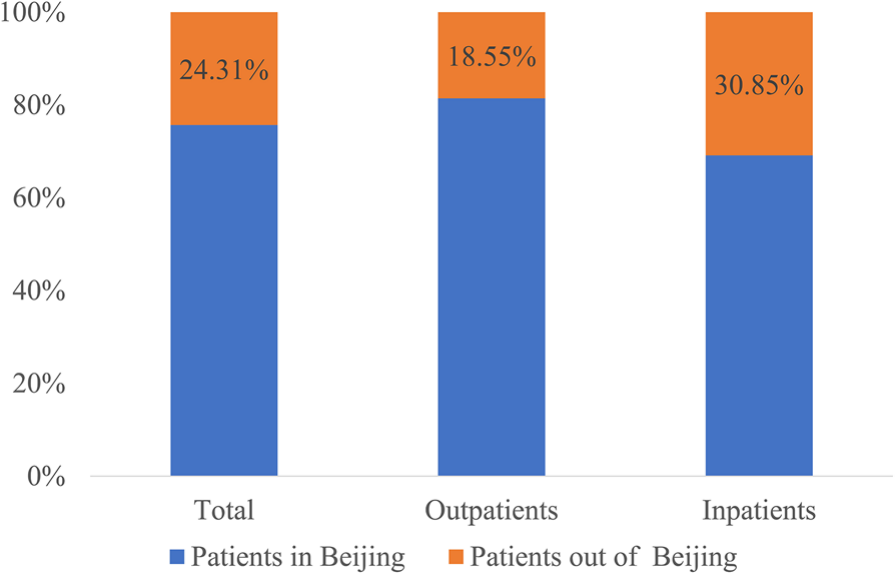
Composition of curative care expenditure (CCE) for patients in different regions

The CCE of medical institutions in Beijing was ¥246.93 billion in 2019, which accounted for 84.01% of the total health expenditure. Patients who came to Beijing from other regions consumed 24.13% of Beijing's CCE (¥60.04 billion). The proportion of CCE for patients outside of Beijing was higher in inpatients than in outpatients (30.85% > 18.55%; see Fig. 1).

#### Composition of CCE in different genders

**Fig. 2:**
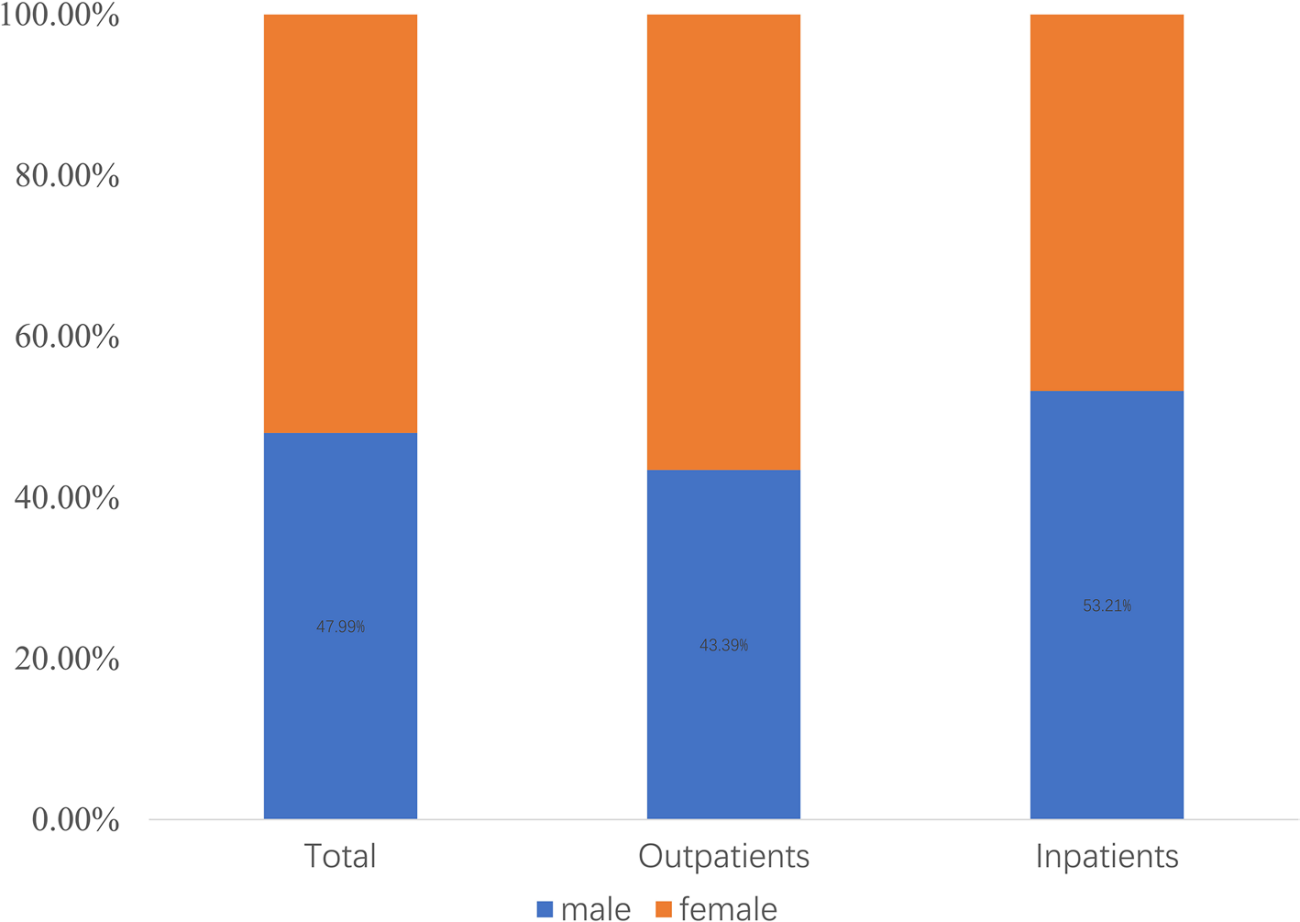
Composition of CCE for different genders of patients

The CCE of male patients was ¥118.51 billion, accounting for 47.99% of the total CCE. The CCE of male patients accounted for 43.39% of outpatients (¥56.95 billion), while that proportion of inpatients was 53.21% (¥61.56 billion; Fig. 2).

#### Composition of CCE according to different age groups

**Fig. 3:**
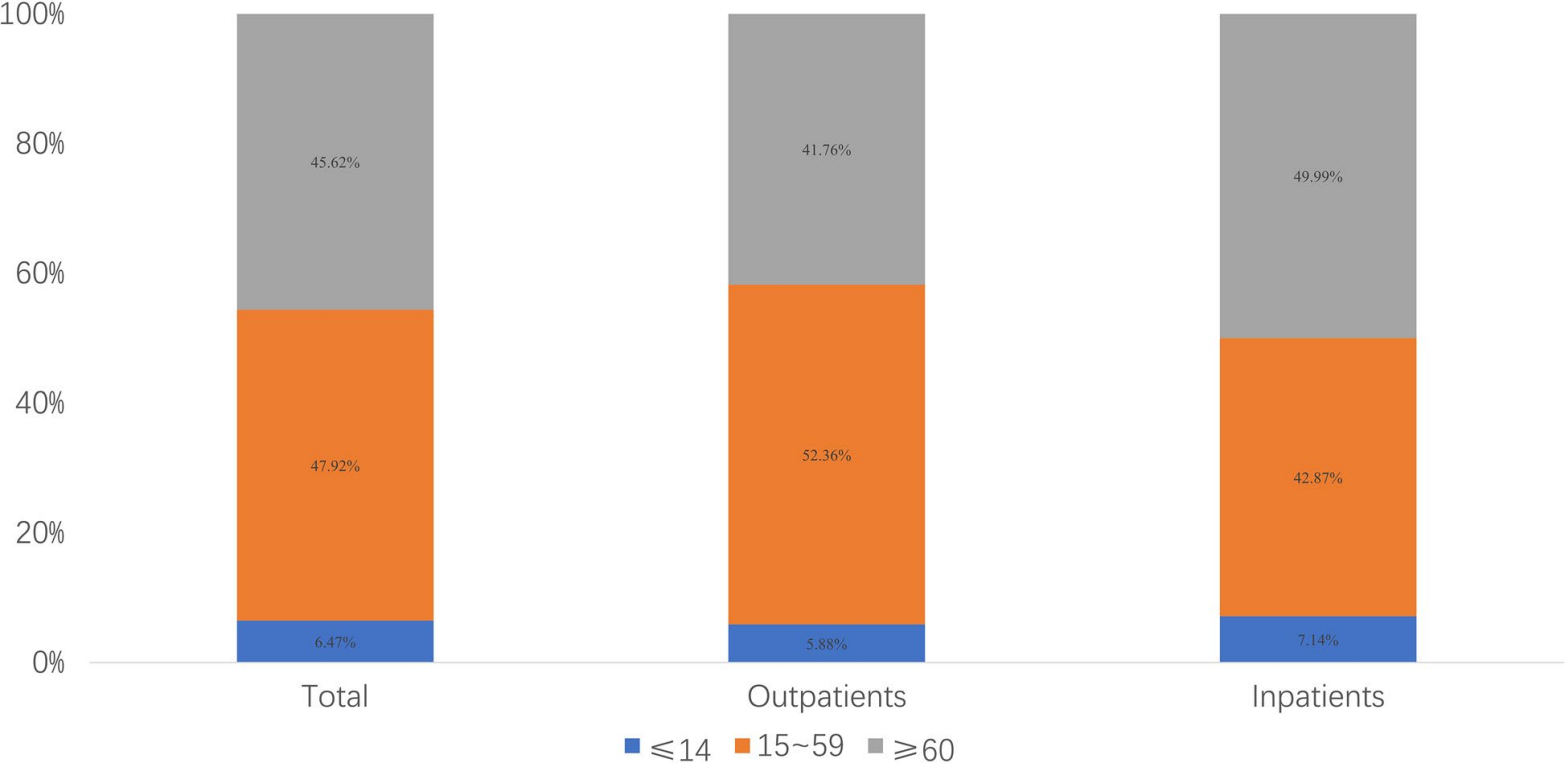
Composition of CCE of patients at different ages

The CCEs of patients in medical institutions younger than 14 years (including those 14 years of age), 15–59 years old, and over 60 years old were ¥15.97 billion, ¥118.32 billion, and ¥112.64 billion, accounting for 6.47%, 47.92%, and 45.62% of the total CCE, respectively. The CCE of patients aged 15–59 accounted for the highest proportion of outpatients, while the CCE of patients over 60 accounted for the highest proportion of inpatients (52.36% vs 49.99%, respectively; Fig. 3).

#### Distribution of CCE according to different classifications

**Fig. 4:**
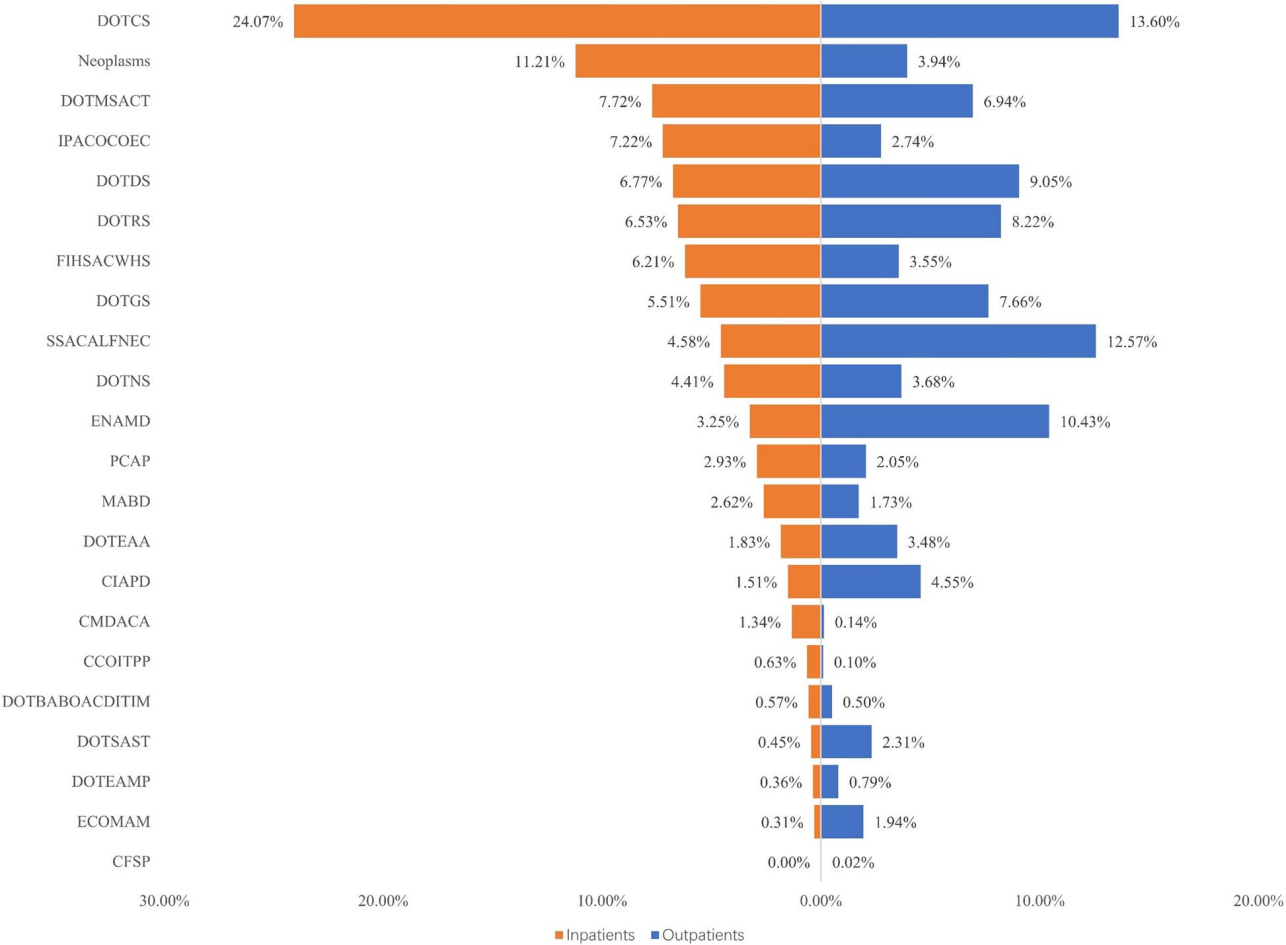
Distribution of CCE for patients according to different classifications DOTCS = Diseases of the circulatory system; DOTMSACT = Diseases of the musculoskeletal system and connective tissue; DOTDS = Diseases of the digestive system; DOTRS = Diseases of the respiratory system; DOTGS = Diseases of the genitourinary system; SSACALFNEC = Symptoms, signs, abnormal clinical and laboratory findings, not elsewhere classified; DOTNS = Diseases of the nervous system; FIHSACWHS = Factors influencing health status and contact with health services; ENAMD = Endocrine, nutritional and metabolic diseases; IPACOCOEC = Injury, poisoning and certain other consequences of external causes; PCAP = Pregnancy, childbirth and puerperium; DOTEAA = Diseases of the eye and adnexa; MABD = Mental and behavioral disorders; CIAPD = Certain infectious and parasitic diseases; DOTBABOACDITIM = Diseases of the blood and blood-forming organs and certain disorders involving the immune mechanism; DOTSAST = Diseases of the skin and subcutaneous tissue; DOTEAMP = Diseases of the ear and mastoid process; ECOMAM = External causes of morbidity and motality; CCOITPP = Certain conditions originating in the perinatal period; CMDACA = Congenital malformations, deformations and chromosomal abnormalities; CFSP = Codes for special purposes

For both outpatients and inpatients, the CCE of diseases of the circulatory system (DOTCS) accounted for the highest proportion, which was 13.60% (¥17.85 billion) and 24.07% (¥27.85 billion), respectively. In addition, for outpatients, the proportions of CCE in symptoms, signs, abnormal clinical and laboratory findings, not elsewhere classified (SSACALFNEC), and endocrine, nutritional, and metabolic diseases (ENAMD) were both also over 10%. Among inpatients, the expenditure on neoplasms was ¥12.97 billion, accounting for 11.21% of the total CCE. Furthermore, the proportions of CCE for diseases of the skin and subcutaneous tissue (DOTSAST), diseases of the digestive system (DOTDS), diseases of the musculoskeletal system and connective tissue (DOTMSACT), and diseases of the genitourinary system (DOTGS) were all at a relatively high level for both outpatients and inpatients. The proportions of CCE in other diseases groups were relatively low (Fig. 4).

### Distribution of CCE according to region, gender, age, and disease classification at different levels and types of medical institutions

#### Distribution of CCE at different institutions by regions

The CCE of patients in and outside of Beijing was ¥86.84 billion and ¥50.34 billion, respectively, accounting for the largest proportion of the flow to tertiary hospitals (46.47% vs 83.84%). A total of 38.42% of CCE (¥71.80 billion) of patients in Beijing and 16.16% of CCE (¥9.70 billion) of patients outside Beijing flowed to secondary hospitals. The proportion of CCE for patients in Beijing that flowed to community hospitals was the lowest, with only about 15.11% (¥28.25 billion).

**Fig. 5:**
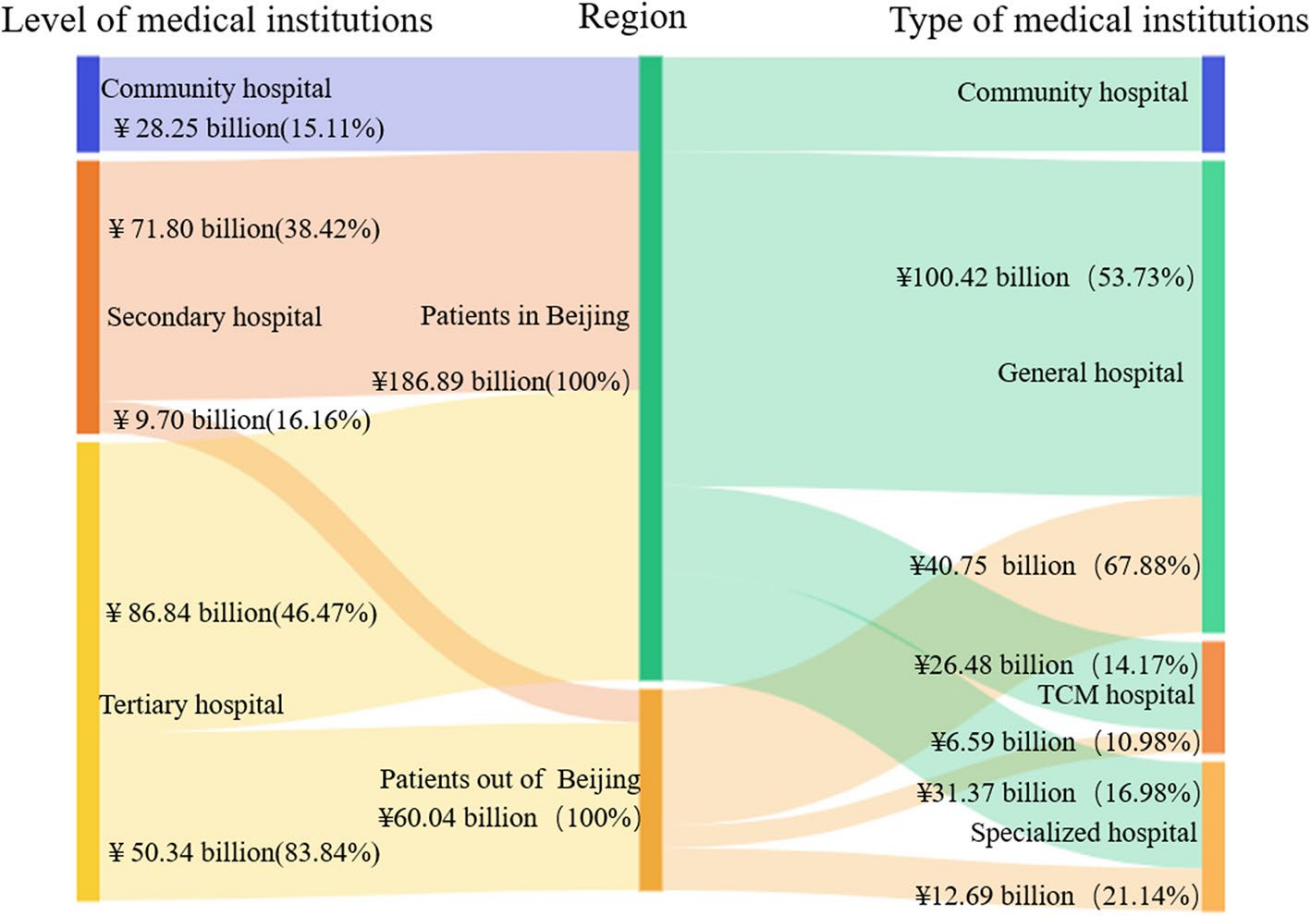
Distribution of CCE according to different institutions by regions

From the perspective of the institutional category, the CCE of both patients in Beijing and patients outside of Beijing mainly flowed to general hospitals with the largest proportion (53.73% vs 67.88%). Compared with patients in Beijing, the CCE of patients outside of Beijing flowing to specialized hospitals accounted for a larger proportion (16.98% vs 21.14%; Fig. 5).

#### Distribution of CCE at different institutions by genders

**Fig. 6:**
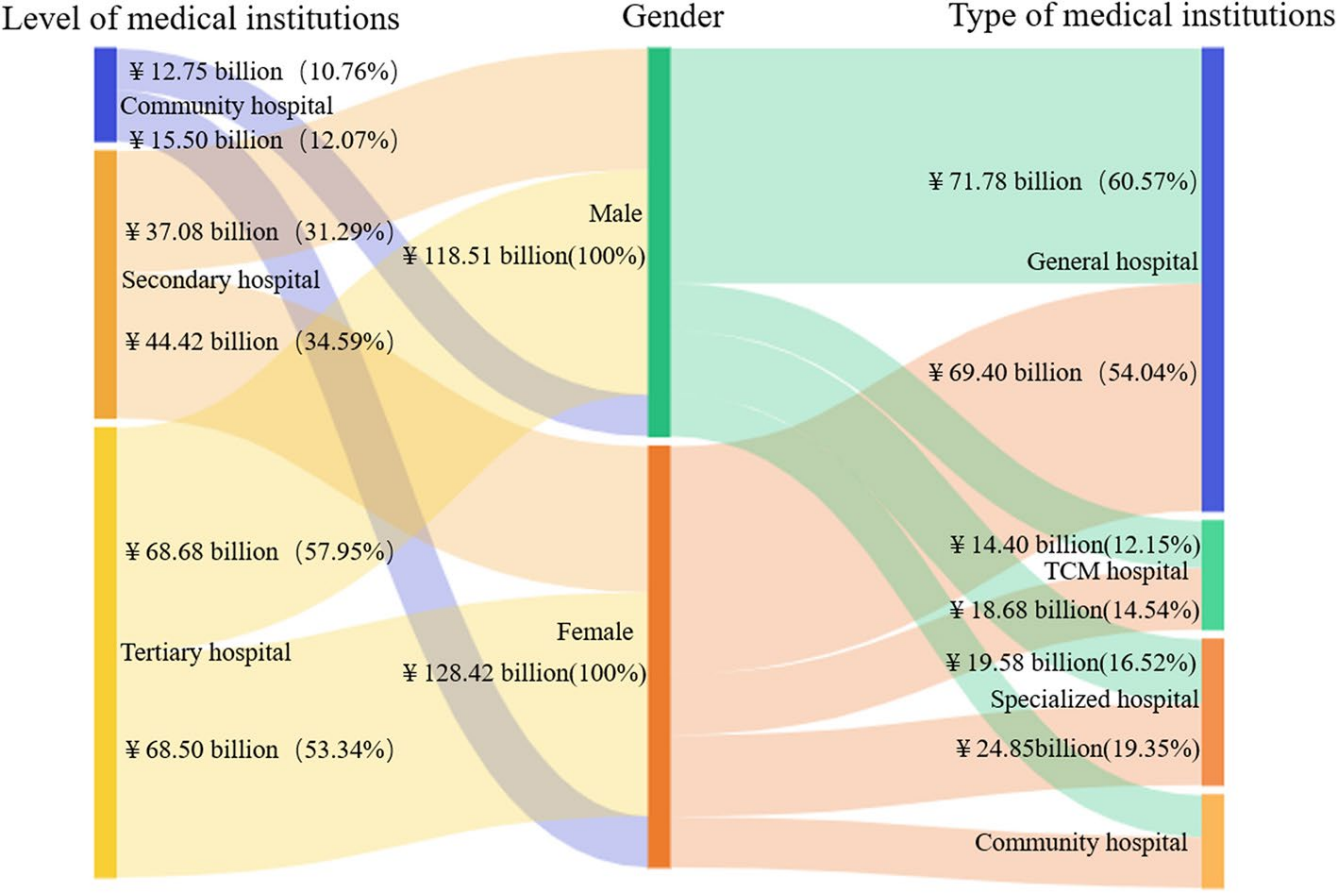
Distribution of CCE according to different institutions by gender

The CCEs of males and females flowing to tertiary hospitals were ¥68.68 billion and ¥68.50 billion, accounting for 57.95% and 53.34% of the total CCE, respectively. Totals of ¥37.08 billion and ¥44.42 billion of their expenditure flowed to secondary hospitals, respectively, both accounting for more than 30%. The proportion of CCE flowing into community hospitals of females was higher than that of males (12.07% vs 10.76%, respectively). More than 50% of the CCE of both male and female patients flowed to general hospitals, and the proportion of male patients was slightly higher than that of female patients. However, the CCE proportions of females flowing to TCM hospitals and specialized hospitals were relatively higher than those of males (Fig. 6).

#### Distribution of CCE at different institutions by ages

Most of the CCE across all age groups flowed to tertiary hospitals, among which the age group over 60 accounted for the lowest proportion of 50.97% (¥57.41 billion). All age groups accounted for more than 30% of the CCE of secondary hospitals, and the age group of patients younger than 14 years accounted for the highest proportion of 39.34% (¥6.28 billion). The proportion of CCE flowing to community hospitals was the smallest across all age groups, among which patients younger than 14 years accounted for the smallest proportion (2.59%/¥0.41 billion), and patients older than 60 years accounted for the largest proportion (15.91%/¥17.92 billion).

**Fig. 7:**
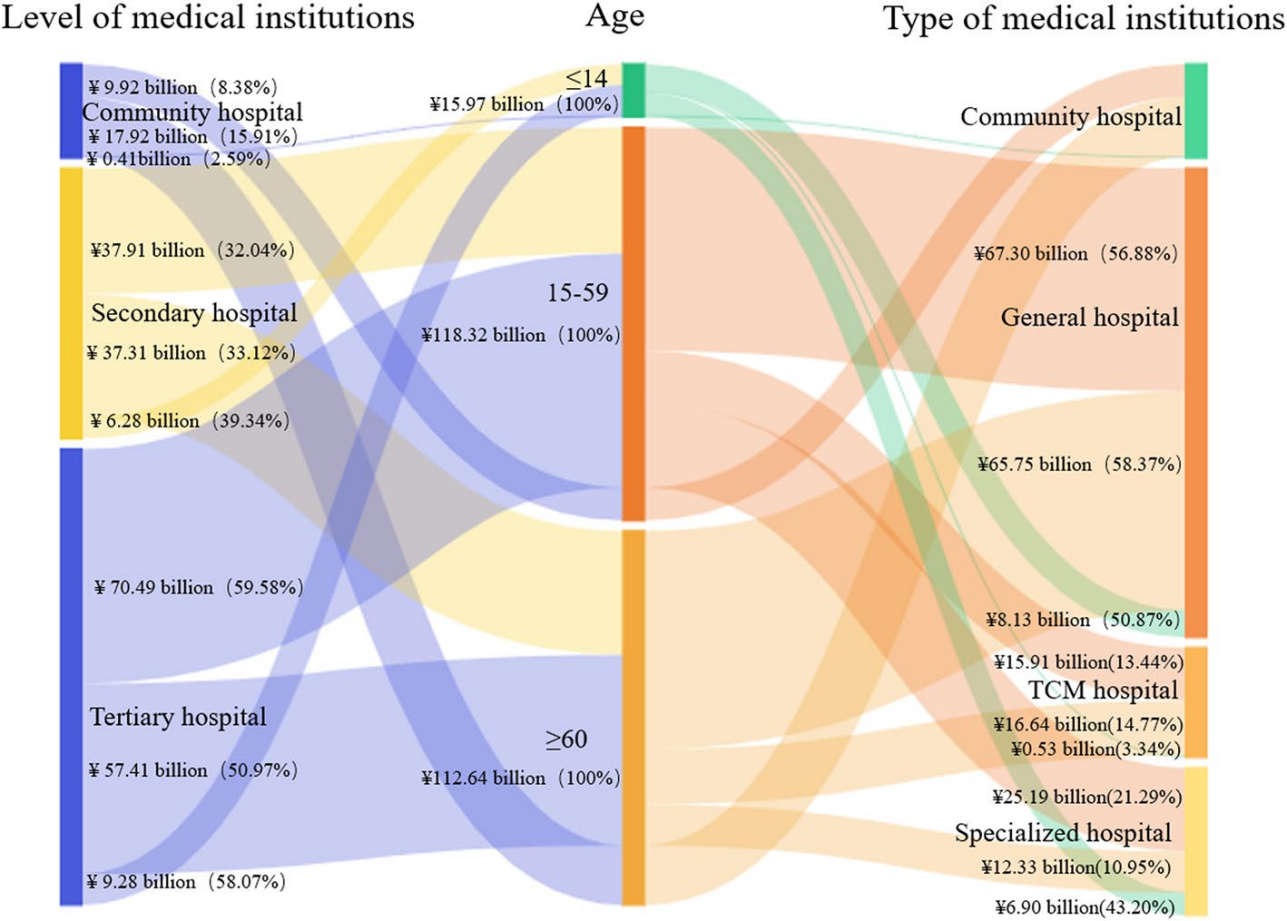
Distribution of CCE according to different institutions by ages

From the perspective of institutional category, the largest proportion of CCE in each age group flowed to general hospitals, and the proportion of CCE flowing to other institutions varied among age groups. For patients younger than 14 years, 43.20% of CCE (¥6.90 billion) flowed to specialty hospitals, while the proportions of CCE flowing to community hospitals and TCM hospitals were less than 4% (¥0.41 billion and ¥0.53 billion, respectively). For patients older than 60 years, the expenditures flowing to TCM hospitals and specialized hospitals were ¥16.64 billion and ¥12.33 billion, accounting for 14.77% and 10.95%, respectively. In comparison, patients aged 15–59 accounted for a larger proportion of the CCE flowing to specialized hospitals, which accounted for 21.29% (¥25.19 billion; Fig. 7).

#### Distribution of CCE at different institutions by diseases

In general, more than 50% of CCE for most disease types flowed to tertiary hospitals. Among them, neoplasms, diseases of the eye and adnexa, congenital malformations, deformations and chromosomal abnormalities, factors influencing health status and contact with health services, and codes for special purposes flowing to tertiary hospitals accounted for more than 70%, some reaching as much as 90%. The CCE of pregnancy, childbirth, and puerperium, as well as certain conditions originating in the perinatal period, accounted for more than 50% of secondary hospitals. A total of 37.20% (¥64.90 billion) of CCE of endocrine, nutritional, and metabolic diseases flowed to community hospitals.

More than 70% of CCE of diseases of the eye and adnexa, diseases of the ear and mastoid processes, diseases of the circulatory system, congenital malformations, deformations and chromosomal abnormalities, as well as codes for special purposes flowed to general hospitals. More than 60% of CCE of mental and behavioral disorders, pregnancy, childbirth and puerperium, as well as certain conditions originating in the perinatal period flowed to specialized hospitals. The proportions of CCE of certain infectious and parasitic diseases, diseases of the skin and subcutaneous tissue, and external causes of morbidity and mortality flowing to TCM hospitals were 40.03% (¥30.90 billion), 33.80% (¥12.01 billion), and 28.98% (¥8.42 billion), respectively; Table 1).

**Table1:** Distribution of curative care expenditure (CCE) for different diseases by the level and type of institutions (¥ billion (%))

	Level of medical institutions				Type of medical institutions				
	Community hospital	Secondary hospital	Tertiary hospital	Total	General hospital	TCM hospital	specialized hospital	Community hospital	Total
CIAPD	5.14(6.65)	32.60(42.24)	39.45(51.11)	77.18(100.00)	15.87(20.57)	30.90(40.03)	25.28(32.75)	5.14(6.65)	77.18(100.00)
Neoplasms	0.66(0.36)	42.51(23.45)	138.14(76.19)	181.30(100.00)	86.47(47.69)	13.07(7.21)	81.10(44.73)	0.66(0.36)	181.30(100.00)
DOTBABOACDITIM	1.39(10.59)	3.62(27.49)	8.15(61.92)	13.17(100.00)	7.00(53.14)	1.35(10.28)	3.42(25.99)	1.39(10.59)	13.17(100.00)
ENAMD	64.90(37.20)	53.98(30.94)	55.57(31.85)	174.45(100.00)	78.22(44.84)	23.00(13.18)	8.32(4.77)	64.90(37.20)	174.45(100.00)
MABD	1.18(2.23)	16.23(30.62)	35.60(67.15)	53.01(100.00)	5.89(11.11)	1.66(3.13)	44.28(83.52)	1.18(2.23)	53.01(100.00)
DOTNS	19.61(19.73)	29.74(29.92)	50.04(50.35)	99.39(100.00)	62.96(63.35)	9.67(9.73)	7.14(7.19)	19.61(19.73)	99.39(100.00)
DOTEAA	2.64(3.94)	11.54(17.28)	52.63(78.78)	66.81(100.00)	56.74(84.93)	3.53(5.28)	3.91(5.85)	2.64(3.94)	66.81(100.00)
DOTEAMP	0.54(3.71)	4.26(29.16)	9.81(67.12)	14.61(100.00)	11.46(78.43)	2.07(14.19)	0.54(3.67)	0.54(3.71)	14.61(100.00)
DOTCS	52.18(11.42)	131.67(28.81)	273.11(59.77)	456.96(100.00)	336.20(73.57)	55.82(12.21)	12.76(2.79)	52.18(11.42)	456.96(100.00)
DOTRS	23.53(12.82)	75.61(41.21)	84.35(45.97)	183.48(100.00)	109.81(59.85)	27.35(14.91)	22.79(12.42)	23.53(12.82)	183.48(100.00)
DOTDS	14.36(7.29)	76.21(38.67)	106.51(54.04)	197.08(100.00)	102.93(52.23)	28.88(14.65)	50.91(25.83)	14.36(7.29)	197.08(100.00)
DOTSAST	4.19(11.79)	13.57(38.17)	17.79(50.04)	35.54(100.00)	15.85(44.58)	12.01(33.80)	3.49(9.83)	4.19(11.79)	35.54(100.00)
DOTMSACT	19.88(11.02)	63.83(35.39)	96.64(53.59)	180.35(100.00)	120.03(66.56)	34.58(19.18)	5.85(3.25)	19.88(11.02)	180.35(100.00)
DOTGS	4.50(2.74)	70.71(43.06)	88.99(54.20)	164.20(100.00)	99.61(60.66)	25.61(15.60)	34.48(21.00)	4.50(2.74)	164.20(100.00)
PCAP	0.03(0.05)	32.62(53.68)	28.12(46.27)	60.77(100.00)	17.46(28.73)	2.13(3.50)	41.15(67.71)	0.03(0.05)	60.77(100.00)
CCOITPP	0.08(0.89)	4.35(50.72)	4.15(48.40)	8.58(100.00)	1.54(17.91)	1.12(13.08)	5.85(68.13)	0.08(0.89)	8.58(100.00)
CMDACA	0.05(0.31)	1.36(7.90)	15.84(91.79)	17.26(100.00)	12.78(74.06)	0.25(1.46	4.17(24.17)	0.05(0.31)	17.26(100.00)
SSACALFNEC	57.42(26.35)	60.57(27.80)	99.89(45.85)	217.88(100.00)	114.92(52.75)	19.66(9.02)	25.88(11.88)	57.42(26.35)	217.88(100.00)
IPACOCOEC	4.14(3.46)	45.77(38.29)	69.63(58.25)	119.54(100.00)	89.37(74.76)	21.30(17.82)	4.73(3.96)	4.14(3.46)	119.54(100.00)
ECOMAM	4.93(16.99)	11.64(40.07)	12.47(42.94)	29.04(100.00)	8.32(28.66)	8.42(28.98)	7.37(25.37)	4.93(16.99)	29.04(100.00)
FIHSACWHS	1.12(0.94)	32.63(27.57)	84.63(71.49)	118.38(100.00)	58.08(49.06)	8.36(7.06)	50.82(42.93)	1.12(0.94)	118.38(100.00)
CFSP	0.01(3.99)	0.00(0.22)	0.27(95.80)	0.28(100.00)	0.26(91.19)	0.01(4.14)	0.00(0.69)	0.01(3.99)	0.28(100.00)
Total	282.48(11.44)	815.03(33.01)	1371.77(55.55)	2469.28(100.00)	1411.79(57.17)	330.76(13.39)	444.25(17.99)	282.48(11.44)	2469.28(100.00)

Endocrine, nutritional, and metabolic diseases, diseases of the circulatory system, and symptoms, signs, abnormal clinical and laboratory findings, not elsewhere classified accounted for relatively large proportions of CCE in community hospitals, each accounting for about 20%. In both secondary and tertiary hospitals, diseases of the circulatory system accounted for a relatively large proportion of CCE, accounting for 16.16% and 19.91%, respectively. In tertiary hospitals, CCE of neoplasms also accounted for 10.07%.

In general hospitals, the CCE of diseases of the circulatory system accounted for 23.81%, while other diseases accounted for a relatively small proportion. Among the CCE of TCM hospitals, certain infectious and parasitic diseases, diseases of the circulatory system, and diseases of the musculoskeletal system and connective tissue accounted for relatively large proportions, each accounting for about 10%. In specialized hospitals, neoplasms, mental and behavioral disorders, diseases of the digestive system, pregnancy, childbirth and puerperium, as well as factors influencing health status and contact with health services accounted for a relatively large amount of CCE, with a cumulative proportion of more than 60% (Table 2).

**Table 2:** Distribution of disease CCE according to different levels and types of institutions (%)

	Level of medical institutions			Type of medical institutions			
	Community hospital	Secondary hospital	Tertiary hospital	General hospital	TCM hospital	specialized hospital	Community hospital
CIAPD	1.82	4.00	2.88	1.12	9.34	5.69	1.82
Neoplasms	0.23	5.22	10.07	6.12	3.95	18.26	0.23
DOTBABOACDITIM	0.49	0.44	0.59	0.50	0.41	0.77	0.49
ENAMD	22.98	6.62	4.05	5.54	6.95	1.87	22.98
MABD	0.42	1.99	2.60	0.42	0.50	9.97	0.42
DOTNS	6.94	3.65	3.65	4.46	2.92	1.61	6.94
DOTEAA	0.93	1.42	3.84	4.02	1.07	0.88	0.93
DOTEAMP	0.19	0.52	0.71	0.81	0.63	0.12	0.19
DOTCS	18.47	16.16	19.91	23.81	16.87	2.87	18.47
DOTRS	8.33	9.28	6.15	7.78	8.27	5.13	8.33
DOTDS	5.08	9.35	7.76	7.29	8.73	11.46	5.08
DOTSAST	1.48	1.66	1.30	1.12	3.63	0.79	1.48
DOTMSACT	7.04	7.83	7.04	8.50	10.46	1.32	7.04
DOTGS	1.59	8.68	6.49	7.06	7.74	7.76	1.59
PCAP	0.01	4.00	2.05	1.24	0.64	9.26	0.01
CCOITPP	0.03	0.53	0.30	0.11	0.34	1.32	0.03
CMDACA	0.02	0.17	1.16	0.91	0.08	0.94	0.02
SSACALFNEC	20.33	7.43	7.28	8.14	5.94	5.83	20.33
IPACOCOEC	1.47	5.62	5.08	6.33	6.44	1.07	1.47
ECOMAM	1.75	1.43	0.91	0.59	2.54	1.66	1.75
FIHSACWHS	0.40	4.00	6.17	4.11	2.53	11.44	0.40
CFSP	0.00	0.00	0.02	0.02	0.00	0.00	0.00
Total	100.00	100.00	100.00	100.00	100.00	100.00	100.00

## Discussion

To the best of our knowledge, this is the first study to explore how the CCE of medical institutions in Beijing was consumed by patients according to their region of origin, gender, age, and disease classification. Different choices of medical institutions for patients in different regions, genders, ages, and disease classifications were analyzed.

In Beijing, medical institutions provide medical services for a large number of patients from other regions. Most patients who travel to Beijing to seek high-quality medical services mainly go to tertiary hospitals. Beijing has rich high-quality medical resources compared with other regions. In 2020, Beijing had 106 tertiary hospitals, with 0.05 tertiary hospitals per 10,000 residents and 12.88 health technicians per 1,000 residents, representing the highest level in China [19, 20]. This phenomenon of off-site medical care promoted the development of medical technology in Beijing to better serve local patients; however, it may also crowd out medical resources for local residents [32, 33]. With the development of high-speed railways and other modes of transportation in China, it has become increasingly convenient for patients from other regions to seek high-quality medical resources in Beijing. To allocate medical resources scientifically, when planning health resources, policy makers at all levels should not only consider the medical needs of local residents, but also those of the residents of other regions. At the same time, the medical insurance department should strengthen cross-regional cooperation and strive to improve the efficiency of medical reimbursement in different places.

Compared with males, females consumed more CCE. Previous research showed that women reported greater morbidity and made greater use of healthcare services [31, 34]. However, females face a greater risk of medical inequality because of economic circumstances and therefore, they focus more on self-care and preventive services [35]. This may be the reason why females used more outpatient services and the proportion of CCE flowed more to community hospitals and secondary hospitals compared with males. The health service needs of females should always receive more attention [36–38]. The gender difference in CCE also suggests that policymakers should enhance health education for males to focus on the early prevention of diseases [39].

Nearly half of the total CCE (45.62%) was consumed by patients older than 60 years. The proportion of CCE for these patients among residents in Beijing was even as high as 50.38%. However, the proportion of permanent residents older than 60 years only accounted for 19.10%, which means that less than one-fifth of the elderly population consumed half of the total CCE [20]. The proportion of the aging population in Beijing has gradually increased from 12.5% in 2010 to 19.60% in 2020 [20, 40]. As China's aging process continues to accelerate, it is foreseeable that an ever larger proportion of the elderly population will consume ever more medical resources in the future. Therefore, a series of measures should be taken to prevent the excessive increase of treatment costs for elderly patients, which is also key to preventing the excessive consumption of medical and health resources in Beijing. Firstly, health education for the elderly should be strengthened and healthy behavioral lifestyles should be established, which is also known as healthy aging [41, 42]. Secondly, the construction of the HMS should be strengthened by addressing the needs of the elderly population. The capacity of geriatric services and health management in community hospitals should be improved to guide elderly patients with common diseases to seek medical treatment at the primary level [30, 43–45]. Thirdly, the payment method of medical insurance should be reformed to meet the demand of elderly patients which can also help to control the excessive increase of medical expenses [46, 47].

Adolescent patients younger than 14 years mainly chose secondary hospitals or tertiary hospitals for treatment, and the proportion of CCE flowing to community hospitals was only 2.59%. The main reason was a lack of pediatricians and professional skills in community hospitals. The lack of pediatric service capabilities has caused a large number of patients with pediatric diseases to choose secondary and tertiary hospitals for treatment [48–50]. This inevitably increases the CCE for adolescents, which to a certain extent increases the burden of access for adolescent patients. With the implementation of the "three-child policy", it has become important to focus on the health of teenagers and make the distribution of CCE for adolescents more reasonable.

It has been proven that the excessive growth of CCE can be controlled by establishing reasonable HMS and guiding patients to choose primary medical institutions [51, 52]. Improving the service capacity of primary medical institutions represented by community hospitals can guide patients to effectively seek medical treatment in an orderly manner. Since Beijing's reform of 2017, measures targeting community hospitals such as unifying the drug catalogs of tertiary hospitals, expanding the range of medical insurance reimbursement, and extending the prescription time for certain chronic diseases have attracted more elderly patients to community hospitals [53, 54]. On the one hand, patients with chronic non-communicable diseases or elderly patients were the main population served by community institutions. In community hospitals, the CCE of chronic non-communicable diseases such as endocrine, nutritional, and metabolic diseases, symptoms, signs, abnormal clinical and laboratory findings, not elsewhere classified, and diseases of the circulatory system accounted for 22.98%, 20.33%, and 18.47% of CCE, respectively. A total of 15.91% of CCE in elderly patients aged 60 years and older was consumed in community hospitals, which was the highest level among all age groups. Previous studies have shown that the elderly were indeed more willing to seek community health service institutions for treatment [55, 56]. On the other hand, the higher proportion of CCE for female patients in community hospitals to a certain extent reflected that community hospitals can better meet the medical needs of female patients in terms of lower prices and their emphasis on prevention [57].

As the leading primary medical institutions in Beijing, community hospitals can not only effectively control the rapid growth of CCE, but also disseminate knowledge about the risk factors of chronic diseases and provide medicine and health counseling to help patients develop healthy living and working habits. However, the technical level and quality of medical services remained influencing factors restricting the flow of more patients to community hospitals [58]. Therefore, various measures should be adopted to improve the service capacity of community hospitals with the goal to further promote hierarchical diagnosis and treatment. On the one hand, the government should strengthen the capacity to provide pediatric services in community hospitals, especially the prevention and treatment of common pediatric diseases such as respiratory diseases, so that community hospitals can attract more adolescent patients in the future [59]. Furthermore, chronic non-communicable diseases consumed the most of CCE. It has been proven that most chronic non-communicable diseases, such as digestive diseases and respiratory diseases, can be prevented and that these preventive measures are cost effective [50, 60]. Policy makers should focus more on the preventive function of community hospitals and invest more in areas such as the prevention and management of chronic non-communicable diseases to better control the growth of CCE by reducing the incidence of disease.

This study showed that the utilization of TCM services in Beijing was not sufficient. As a traditional treatment service in China, TCM services have been benefiting from the advantages of safety, simplicity, and affordability. However, TCM hospitals—the main providers of TCM services—currently account for a relatively low percentage of CCE in Beijing (13.39%), which is much lower than that of general hospitals (57.17%) and specialty hospitals (17.99%). The goal of significantly increasing the capacity of TCM health services as proposed in “China's Strategic Planning Outline for TCM Development (2016–2030)” has not yet been reached. The utilization of TCM services by adolescent patients and patients with chronic non-communicable diseases was insufficient. Male patients also make less use of TCM services compared to female patients [60, 61]. With the promotion of the "Healthy China Plan", the following measures can further promote the development of TCM services. First, the government should formulate policies to support the development of dominant diseases of TCM and increase the medical reimbursement rate for TCM services to guide more patients with chronic diseases to utilize TCM services [62–64]. Second, TCM publicity and education should be increased especially for the youth and male population. The influence of TCM services among specific groups can be enhanced through the promotion and education of TCM knowledge in primary and secondary school classes and at the community level. Furthermore, measures such as changing the dosage forms and employing methods of Chinese herbal medicines should be taken to improve the utilization of Chinese medicine services [65, 66].

### Limitations

Patient data from central hospitals were not accessible mainly for reasons of affiliation; therefore, central hospitals were not included in the sample. However, the structures and characteristic specialty profiles of patients of the central hospitals located in Beijing are similar to those of municipal hospitals. Therefore, using patient data from municipal hospitals as a sample could also reflect the situation in central hospitals to a certain extent. Additionally, CCE of outpatient institutions was not included in this study. On the one hand, the data of these outpatient institutions were not sufficiently accurate due to the lack of well-established information systems and the need of privacy. On the other hand, this part of CCE accounted for so small proportion (less than 5%) that had less possibility to affect the conclusion.

## Conclusions

This study used SHA 2011 to explore utilization disparities of the CCE in medical institutions in Beijing by different regions, genders, ages, and disease classifications. The results suggest that more consideration should be given to the growing medical service needs of elderly patients and patients with chronic diseases, while the demand of non-local patients for high-quality medical services should not be overlooked in the future allocation of resources. The government should strengthen the construction of service capacity and medical quality of community-level medical institutions to guide patients, especially adolescents with common diseases and patients with chronic diseases, to flow to community hospitals. This strategy is of great significance for improving HMS and controlling the rapid growth of CCE.

## Data Availability

All data generated or analysed during this study are included in this published article.

## Abbreviations

CCE: Curative care expenditure
HMS: Hierarchical medical system
SHA 2011: System of Health Accounts 2011
DOTCS: Diseases of the circulatory system
DOTMSACT: Diseases of the musculoskeletal system and connective tissue
DOTDS: Diseases of the digestive system
DOTRS: Diseases of the respiratory system
DOTGS: Diseases of the genitourinary system
SSACALFNEC: Symptoms, signs, abnormal clinical and laboratory findings, not elsewhere classified
DOTNS: Diseases of the nervous system
FIHSACWHS: Factors influencing health status and contact with health services
ENAMD: Endocrine, nutritional and metabolic diseases
IPACOCOEC: Injury, poisoning and certain other consequences of external causes
PCAP: Pregnancy, childbirth and puerperium
DOTEAA: Diseases of the eye and adnexa
MABD: Mental and behavioral disorders
CIAPD: Certain infectious and parasitic diseases
DOTBABOACDITIM: Diseases of the blood and blood-forming organs and certain disorders involving the immune mechanism
DOTSAST: Diseases of the skin and subcutaneous tissue
DOTEAMP: Diseases of the ear and mastoid process
ECOMAM: External causes of morbidity and mortality
CCOITPP: Certain conditions originating in the perinatal period
CMDACA: Congenital malformations, deformations and chromosomal abnormalities
CFSP: Codes for special purposes
